# Simultaneous Sensing and Actuating Capabilities of a Triple-Layer Biomimetic Muscle for Soft Robotics

**DOI:** 10.3390/s23229132

**Published:** 2023-11-12

**Authors:** Francisco García-Córdova, Antonio Guerrero-González, Joaquín Zueco, Andrés Cabrera-Lozoya

**Affiliations:** 1 Department of Thermal and Fluid Engineering, Polytechnic University of Cartagena, Campus Muralla del Mar, 30203 Cartagena, Spain; francisco.garcia@upct.es (F.G.-C.); joaquin.zueco@upct.es (J.Z.); 2 Department of Automation, Electrical Engineering and Electronic Technology, Polytechnic University of Cartagena, Campus Muralla del Mar, 30203 Cartagena, Spain; 3 Department of Applied Physics and Naval Technology, Polytechnic University of Cartagena, Campus Muralla del Mar, 30203 Cartagena, Spain; andres.cabrera@upct.es

**Keywords:** conducting polymers, biomimetic, artificial muscles, soft actuators, neural control

## Abstract

This work presents the fabrication and characterization of a triple-layered biomimetic muscle constituted by polypyrrole (PPy)-dodecylbenzenesulfonate (DBS)/adhesive tape/PPy-DBS demonstrating simultaneous sensing and actuation capabilities. The muscle was controlled by a neurobiologically inspired cortical neural network sending agonist and antagonist signals to the conducting polymeric layers. Experiments consisted of controlled voluntary movements of the free end of the muscle at angles of ±20°, ±30°, and ±40° while monitoring the muscle’s potential response. Results show the muscle’s potential varies linearly with applied current amplitude during actuation, enabling current sensing. A linear dependence between muscle potential and temperature enabled temperature sensing. Electrolyte concentration changes also induced exponential variations in the muscle’s potential, allowing for concentration sensing. Additionally, the influence of the electric current density on the angular velocity, the electric charge density, and the desired angle was studied. Overall, the conducting polymer-based soft biomimetic muscle replicates properties of natural muscles, permitting simultaneous motion control, current, temperature, and concentration sensing. The integrated neural control system exhibits key features of biological motion regulation. This muscle actuator with its integrated sensing and control represents an advance for soft robotics, prosthetics, and biomedical devices requiring biomimetic multifunctionality.

## 1. Introduction

There is growing interest and significant attention in bio-inspired applications of electroactive materials in robotics due to advances in creating new materials and actuators that mimic both the macroscopic and microscopic behavior of natural muscles. In this context, conjugated polymers (CPs) stand out for their remarkable electrochemical–mechanical properties, which enable them to perform simultaneous sensing and actuation, thus mimicking the bi-functionality characteristic of natural muscles. Conductive polymers achieved notable success in the field of actuators. During their oxidation/reduction process, CP films undergo variations in their volume, which can be exploited to generate linear or angular movements. However, it is important to note that, at present, these materials face challenges related to their efficiency and compliance, which need to be addressed in future research.

When analyzing biological systems, it is observed that each organ is made up of flexible organic materials and operates in a moist environment, as well as being organized in a natural way. Biological processes depend on chemical reactions that take place within living cells. In robotics and medical technology, there is increasing demand for artificial muscles or flexible actuators. In recent years, engineering focused on the creation of materials capable of emulating biological composition, reactions, and functions. Among these materials, reactive gels, which consist of CPs, water, and ions, are particularly noteworthy, serving as the most elementary bio-inspired materials [1,2,3].

CPs, in a diverse spectrum of applications, such as light-emitting diodes, antistatic coatings, electrochemical sensors, batteries, conductive fabrics and textiles, electromagnetic interference protection, supercapacitors, photovoltaics, corrosion protection, information storage devices, actuators known as artificial muscles, biosensors, biomedical devices, and many others, demonstrated their versatility [1,2,3,4,5,6,7,8,9,10,11,12,13,14,15,16,17,18,19,20,21,22,23,24,25,26,27,28,29,30,31,32,33,34,35,36,37,38,39]. This is due to their remarkable electrochemical and physical properties, which contribute greatly to their uniqueness [4,5,6]. The choice of CPs in these various applications is based on their ability to regulate both expansion and contraction of volume, along with conductive properties responding to applied voltage [7]. These volume and electrical conductivity changes are closely related to the electrochemical processes of reduction and oxidation, followed by the expulsion or insertion of anions, respectively. The opposite is the case with cations [8,9].

When immersed in the presence of electrolytes, CP films undergo electrochemical processes that generate changes in their volume [10,11,12,13,14]. Under constant current, according to the rate equation, any modification in the physical or chemical conditions that influences the reaction velocity causes a modification in the electrode voltage, following the principles of electrochemical kinetics. In this context, it is plausible to consider that the variation in the potential of the reactive material functions as a sensor sensitive to the chemical or physical conditions of the environment. Consequently, conductive polymers emerge as materials that exhibit the characteristics of being flexible, operating in wet environments, performing functions as actuators, and also fulfilling the role of sensors [15]. In this way, they manage to emulate most of the properties observed in biological materials.

Among the wide range of available conducting polymers, extensive research focused on polypyrrole (PPy) films, since PPy exhibits electrochemical activity over a broad pH range, from acidic to alkaline, and its production is straightforward. It was confirmed that PPy films, which perform anion or cation exchanges during reactions, can sense changes in temperature and concentration of the surrounding environment [16,17,18,19,20,21,22]. Specifically, triple-layer and bilayer synthetic muscles fabricated from these CP films can detect variations and assess the weight of objects with which they interact [23,24,25,26]. Additionally, tactile muscles capable of identifying obstacles in their path were developed. These devices can supply data regarding an obstacle’s mechanical strength when subjected to pushing or attempted displacement [27,28].

When we delve into the study of artificial muscles based on conducting polymers (CPs) in which cation exchanges predominate during reactions, it is crucial to underline that the volume changes and movements induced by electric current occur in the contrary direction to what is observed with anion-exchanging CPs artificial muscles [26,29,30]. In the case of cation-exchanging CPs materials, they undergo expansion during reduction due to cation influx, whereas they contract during oxidation by expelling cations. In contrast, anion-exchanging CPs materials contract during reduction and expand during oxidation. In the last decade, it was shown that both DBS-doped polypyrrole films capable of cation exchange and artificial bilayer devices constructed with these films attached to a non-conductive tape can sense the physical and chemical variables of the environment while engaged in reactions [31,32,33,34,35,36,37,38,39]. In this context, the device potential is monitored through a reference electrode. However, it is essential to note that this system requires the presence of three electrodes: a working electrode for the actuator, a metal counter electrode, and a reference electrode. This configuration poses practical challenges in device development and leads to significant energy consumption. Most of the energy consumed goes to drive electrolytic discharge at the metal counter electrode, while the energy used by the muscle itself represents a small fraction of total electrical energy consumption.

These devices can be considered examples of soft robots, as they are constructed of flexible and malleable materials that can change shape and move in response to electrical stimuli. Research in the field of soft robotics focuses on the development of robots inspired by natural organisms, using soft, deformable materials instead of rigid components. Artificial muscles mimic the electrochemical and biomechanical properties of natural muscles, enabling them to perform controlled movements and sense physical and chemical variables in their environment. These soft robots based on conductive polymers represent a promising advance for applications such as prosthetics, assistive robots, and biomedical devices, as they combine actuation and sensing capabilities similar to those of living organisms. Ongoing research aims to improve the properties and control of these biomimetic soft robots.

In this study, the preparation, fabrication, and characterization of a triple-layer biomimetic muscle is presented. The biomimetic muscle proposed for this research is composed of three layers of PPy-DBS/non-conductive adhesive tape/PPy-DBS. Simultaneously, the sensory and actuation capabilities of this biomimetic muscle will be analyzed by cycling quadratic voluntary movements by varying the amplitude of the free end of the muscle at ±40°, ±30°, and ±20°. Throughout these voluntary movements, we will explore sensory attributes concerning electrolyte concentration, operating temperature, and applied electric current, employing an aqueous solution of lithium perchlorate (LiClO_4_) as the electrolyte [33]. This electrochemical–mechanical system consists of two polymeric reactive electrodes, one for oxidation and the other for reduction. What makes it even more impressive is its dual ability to function as both actuator and sensor, a quality known as “self-sensing actuators” [33,34]. Thus, these conductive polymer devices can be considered materials that act as flexible, wet actuators and sensors, enabling them to sense environmental conditions in an analogous way to biological muscles [35,36].

Furthermore, a control system for voluntary movement of the three-layer biomimetic muscle is implemented using a neural network inspired by the spinal cord cortex, within the limits of neurophysiology and motor psychophysics. The neural controller sends agonist and antagonist activation signals to each PPy-DBS layer of the biomimetic muscle to achieve voluntary movement control. This study represents progress toward the interaction between biomimetic materials, such as conductive polymers, and understanding the capabilities of neural circuits in the central nervous system structure, similar to living organisms. Moreover, the corticospinal controller’s efficacy is showcased through experimental findings in which the control system has fundamental kinematic characteristics that mimic human motion, while possessing dynamic compensation capabilities.

This paper is based on previous research conducted by García-Córdova et al. in 2011 [33]. In that study, they did not implement an angular position control system; instead, they relied solely on control signals, such as anodic or cathodic currents applied to the actuator in an open loop, and manually adjusted the times to reach the desired positions. In addition, previous research focused exclusively on the analysis of sensory behavior at a specific angular position, without exploring the sensory capability of the actuator in response to varying angular motions to changing chemical and physical conditions of the environment. The authors did not investigate the potential relationship between various currents and the angular velocity, charge density, or desired angle in a trilayer device, to assess the plausibility of self-sensing its position for a closed-loop system. It is worth noting that studies exist related to this topic, involving bilayer devices with a different material type than that presented in this study [24,28].

The novelty presented by this work is the innovation of the integration of a bio-inspired neurocontroller for controlling desired angular movements of a triple-layer biomimetic muscle, similar to what occurs with biological muscles. When controlling a desired position, the neurocontroller sends agonist–antagonist signals (anodic and cathodic currents corresponding to the movement direction) through two wires to the triple layer muscle and determines the time of establishment of the control signal for the desired position. Once the desired angle is reached at the end of the actuator, the neurocontroller stops sending the control signal, ensuring that the actuator stops ejecting or inserting anions or cations, allowing the desired position to be reached under various chemical or physical environmental conditions. The triple-layered biomimetic muscle exhibits the ability to sense and act simultaneously, which differentiates it from previous artificial muscle designs that lacked this integrated functionality. A control system based on a neurobiologically inspired cortical neural network is implemented, which sends agonist and antagonist signals to the conducting polymeric layers of the muscle. This represents a novel approach to the interaction between biomimetic materials and neural control. The integrated sensing and actuation capabilities of the biomimetic muscle represent a significant advance in soft robotics, prosthetics, and biomedical devices pursuing biomimetic functionality.

The structure of this article is as follows: First, Section 2 is devoted to describing the materials used, the fabrication process, and the sensory evaluation of the trilayer biomimetic muscle. In the same section, an innovative bio-inspired control system designed for voluntary movements of the biomimetic muscle is introduced, and the corresponding equations are provided in Appendix A. Section 3 focuses on the presentation of experimental results related to the simultaneous actuation and sensing capabilities of the biomimetic muscle, addressing aspects such as sensed conduction currents, working temperatures, and electrolyte concentrations. The influence of electric current density on angular velocity, electric charge density, and desired angle is also examined, accompanied by a detailed analysis of the results obtained. Section 4 is devoted to discussing the sensing and actuation capabilities of both the triple-layer biomimetic muscle and the corticospinal neural controller used for voluntary movement control. Finally, Section 5 presents conclusions related to the triple-layer biomimetic muscle and its potential applications.

## 2. Materials and Methods

### 2.1. Materials

For the manufacture of the triple-layer artificial muscle, in a first stage, the pyrrole (Py) purification process (98%, Sigma-Aldrich^®^, St. Louis, MO, USA) was carried out by means of vacuum distillation through a membrane vacuum pump (MZ 2C, SCHOTT^®^, New York, NY, USA). It was then stored in a light-free environment at a temperature of −10 °C. Both DBS acid solution (Sigma-Aldrich^®^; 70% by weight in 2-propanol) and anhydrous lithium perchlorate salt (95%, Sigma-Aldrich^®^) were used in their original state, without any modifications. Ultrapure water provided by the Milli-Q^®^ IQ 7000 (Merck KGaA, Darmstadt, Germany) was used throughout the process. The DBS was kept refrigerated at 5 °C.

### 2.2. Electropolymerization of PPy-DBS Films

PPy-DBS films were fabricated under dark conditions, maintaining a constant temperature of 5 ± 1 °C, inside a single-compartment electrochemical cell equipped with three stainless steel (AISI 316) electrodes. In the preparation process, 50 mL of a water solution composed of 0.2 M pyrrole monomer and 0.15 DBS was used. To control the polymerization temperature, a Jubalo^®^ F25-HE cryostat/thermostat (JULABO GmbH, Seelbach, Germany) was used. The working electrode has a surface area of 5 × 2.1 cm on each side and a thickness of 1.24 ± 0.01 mm. For the counter electrodes, two larger electrodes were chosen, with a surface area of 5.5 × 2.5 cm, which were made of the same material as the working electrode. The counter electrodes (CE) were placed symmetrically, maintaining a precise separation of 1 ± 0.15 cm from the working electrode (WE), which ensured a uniformly distributed electric field. Additionally, a standard reference electrode (RE) of Ag/AgCl (3 M KCl) manufactured by Metrohm^®^ (Herisau, Switzerland) was applied in the process.

**Figure 1:**
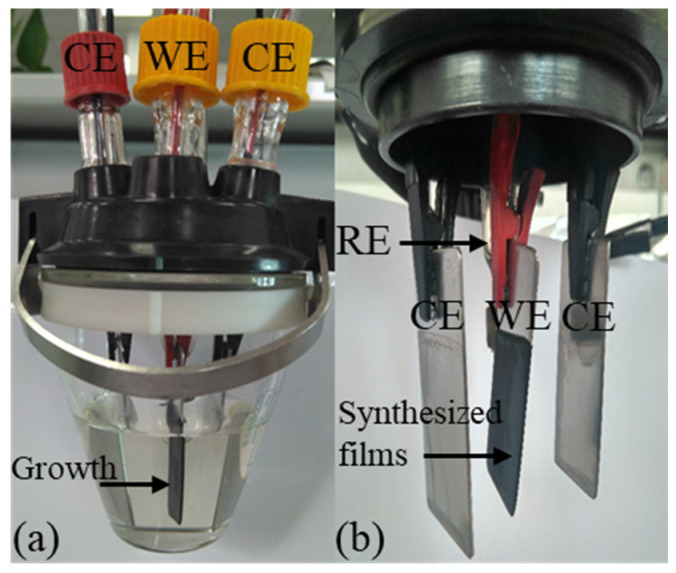
PPy-DBS film synthesized by the galvanostatic electropolymerization process. (**a**) Polymerization of pPy-DBS films grown on stainless steel. (**b**) Final result of the synthesis process. This process was carried out under dark conditions, for a period of 100 min and maintaining a constant anodic current density of 0.8 mA∙cm^−2^ at an ambient temperature of 5 ± 1 °C.

The PPy-DBS films were synthesized by a galvanostatic process, and over a period of 100 min, a constant anodic current density of 0.8 mA cm^−2^ was applied. Polymerization growth was performed on both faces of the submerged portion of the working electrode, which had a surface area of 3.5 × 2.1 cm on each side, as can be seen in Figure 1. The total charge consumed during the electropolymerization process was 64 C. Next, the stainless steel electrode coated with PPy-DBS was again immersed in the same mixture and maintained at a constant electrical voltage of 0.05 V for a period of 240 s. This step was performed with the aim of reaching an intermediate level of oxidation in the material [33,37].

Subsequently, the PPy-DBS film was carefully removed from the edges of the steel electrode. Once detached from the working electrode, the films were subjected to a deionized water washing process and immersed in deionized water for a period of 48 h, all under dark conditions, in order to remove any excess DBS present on the surface of the polymer. Deionized water was changed every 8 h. This procedure was carried out in order to improve the adhesion to the non-conductive tape and ensure the proper performance of the designed devices. PPy-DBS films were subjected to an air-drying process, carried out at room temperature, specifically at 20 ± 1 °C. The mass of the electrogenerated polymer in its dry state was quantified with a precision balance (Sartorious^®^, Göttingen, Germany, Model SE2) with an accuracy of ±0.25 μg, resulting in a mass of 58.13 ± 0.01 mg. From this mass, two individual films were obtained, each covering one of the electrode faces, with 28.17 ± 0.01 mg mass each. These films had a thickness of 37 ± 1 μm, measured with a (COMECTA^®^, Abrera, Barcelona) electronic digital micrometer, and were applied in the subsequent experiments.

### 2.3. Manufacture of the Triple-Layer Biomimetic Actuator

**Figure 2:**
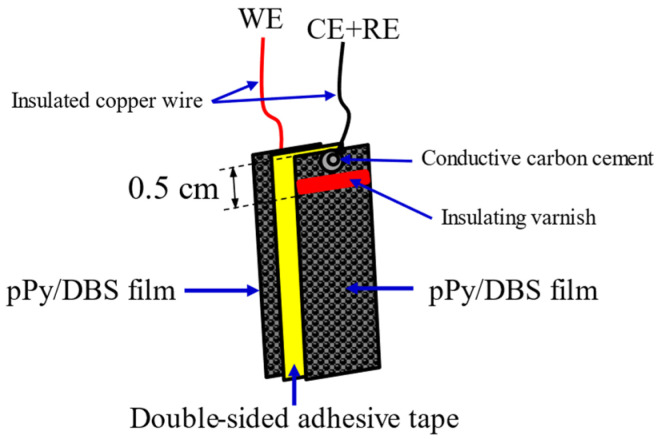
Triple-layer artificial muscle assembly.

The triple-layer device is configured with two PPy-DBS films and an intermediate layer of double-sided adhesive tape, the latter of which is of a nonconductive polymeric nature from 3M, commonly used in the market. Each of the PPy-DBS films are adhered to both sides of the tape, as can be seen in Figure 2. These films, with an intermediate oxidation state, have a weight of 3.4 ± 0.01 mg, a thickness of 37 ± 1 μm, and dimensions of 2 × 0.5 cm each, (these films are the result of cutting the electrogenerated PPy-DBS films into strips) [37]. The conductivity of the PPy-DBS films was 37.11 ± 0.01 S∙cm^−1^ (in this study, 10 measurements of 5 films were made to obtain the average) and determined by the four-point probe method with a surface resistivity meter (VEECO^®^ FPP-5000, Plainview, New York, USA), these results are very close to those obtained in [38]. According to the study by Khanh et al. (2019) [39], the conductivity is improved when electropolymerization is performed at low temperatures.

**Figure 3:**
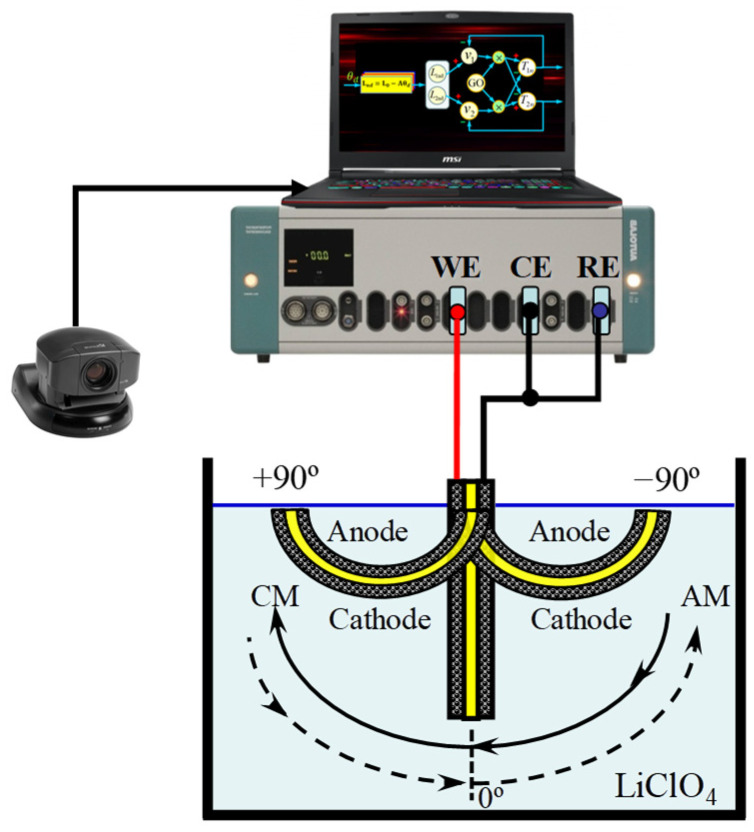
Schematic of the experimental system, including the electrode arrangement and electrochemical vessel (CE, counter electrode; RE, reference electrode; and WE, working electrode). This setup was used to analyze and evaluate the performance (as sensor and actuator) of the biomimetic muscle. CM is the clockwise movement and AM is the anticlockwise movement.

During the process, one of the PPy-DBS films was connected to the WE output of the galvanostat–potentiostat. Meanwhile, the second PPy-DBS film, located at the opposite end of the trilayer device, was linked to the output assigned to the CE of the galvanostat–potentiostat, while creating a direct link to the RE. Importantly, both conductive polymer films are kept electrically isolated by the presence of a non-conductive tape separating them [28], as shown in Figure 3.

In addition, a non-reactive insulating barrier, consisting of a line of MaxFactor^®^ red nail polish with a width of 1.2 ± 0.1 mm, was applied on both sides of the PPy-DBS layers. This strategy was implemented with the aim of preventing potential short circuits in the experiments by inhibiting the upward capillary movement of the aqueous electrolyte solution and its contact with the metallic components situated at the top of the film [37], as illustrated in Figure 2. As part of the experiment, the three-layer artificial muscle assembly was immersed in aqueous electrolyte solutions and subjected to extensive characterization. The muscle angle was controlled by a proposed bio-inspired neurocontroller sending agonist and antagonist electric current signals for each polymer layer and recording the chronopotentiometric responses. The unrestrained end of the trilayer device executes a constant angular motion around its anchor point, as shown in Figure 3.

### 2.4. Sensing Characterization of PPy-DBS Films

Control of the movement of the triple-layered artificial muscle was carried out using Matlab^®^ R2013a software, which is connected to an Autolab PGSTAT 100 galvanostat–potentiostat (Metrohm Autolab^®^, Herisau, Switzerland). This trilayer device is controlled by electrochemical software (NOVA 2.1 from Metrohm Autolab^®^) implemented on a personal computer, as shown in Figure 3. The temperature was kept constant using a Jubalo^®^ F25-HE cryostat/thermostat, which guarantees an accuracy of 0.1 °C. It should be noted that the remaining experiments were performed at room temperature in a range of 20 ± 1 °C.

Experiments for sensing characterization were carried out in a single-compartment electrochemical cell. The three-layered artificial muscle was attached with a non-conductive alligator clip, which featured independent metal contacts installed individually on both sides of the clip. This configuration allowed connections to be established with both the WE film and the CE + RE film of the artificial muscle, allowing precise control of electrical interactions in the device. Control of muscle movement is achieved through a bio-inspired neurocontroller of a simplified cortical network by sending agonist–antagonist actuation signals to the films forming the triple-layer actuator. Measurements, both of an electrochemical and physical nature, were carried out in aqueous solutions with a 0.1 M concentration of LiClO_4_. This provided a consistent and controlled experimental environment for the evaluation of the properties and behavior of the system under study. The polymeric artificial muscle was monitored by constant clockwise (CMs) and anticlockwise (AMs) bending movements, forming angles (θ) of ±20°, ±30°, and ±40°. It was established in this paper that a bending movement cycle is composed of CMs and AMs comprised between a range of motion (e.g., ±40°), where CMs start from −40° to 40°, while AMs from 40° to −40°, in both cases forming a displacement of 80°. Additionally, the units of angle-dependent slopes will be set in radians and angular velocities in rad/s.

The angular velocity was determined by means of a vision system that captured images through a SONY^®^ EVI-D31 digital camera, which was controlled by a Matrox^®^ card. Image analysis was performed using a C++ programmed control system. The data were then processed in Matlab^®^. For a better understanding of the experimental setup, it is presented in detail in Figure 3.

### 2.5. Cortical Neural Network for Triple-Layer Biomimetic Muscle Motion Control

**Figure 4:**
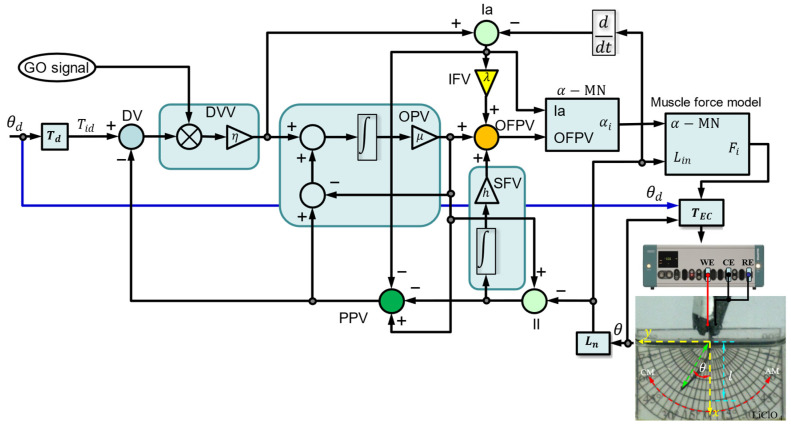
Block diagram of the neurocontroller inspired by a neurophysiological cortical neural network. $T_{id}$, target position vector (TPV); α, alpha motoneuron (α-MN); OFPV, outflow force + position vector; DV, difference vector; IFV, inertial force vector; DVV, desired velocity vector; PPV, perceived position vector; OPV, outflow position vector; GO, scalable gating signal; SFV, static force vector; $L_{in}$, normalized muscle length (virtually); II, type II afferent fiber; Ia, type Ia afferent fiber; $T_{d}$, transformation to the tendon space (virtually); $\theta_{d}$, desired joint angle; $F_{i}$, agonist-antagonist forces; and $T_{EC\;}$, transformation to anodic and cathodic currents. The symbol ∫ represents integration, + excitation, × multiplicative gating, and – inhibition.

The biomimetic muscle was controlled by a bio-inspired neurocontroller inspired by corticospinal pathways in the brain and spinal cord. The proposed circuit connects neurophysiologically identified cell types, including connections to alpha motor neurons and muscle receptors. The corticospinal tract conducts impulses from the brain to the spinal cord, primarily controlling voluntary joint movements through agonist and antagonist muscles. Figure 4 shows the connectivity of the proposed bio-inspired neurocontroller. This approach is based on different neuronal representations of variables encoded through activity levels distributed in different cortical populations.

The proposed neurocontroller acts as a central pattern generator, with the ability to incrementally generate the desired joint movement trajectories. This is achieved by interpolation between the initial and final muscle length controls. This is particularly applicable to synergistic agonist–antagonist muscles, such as the PPy-DBS layers of biomimetic muscle, which contribute to the predefined multi-joint motion. The joint velocity is controlled by the multiplication of two key signals: a volitional activation signal known as the GO signal, which represents the *globus pallidus* [40,41], and a *difference vector* (DV), which continuously monitors the remaining alterations in muscle length required to reach the desired angle ($\theta_{d}$). A detailed description of the system of cortical network control equations is provided in Appendix A.

In the operation of this neural control system, the desired angle for the muscle is transformed into normalized target position signals. These target signals are compared with the actual position to calculate a DV for error correction. While the scalable gate signal (GO) regulates the speed and effort of the movements. The DV is multiplied by the GO to generate a *desired velocity vector* (DVV) that drives the motion. An integrate-and-fire model converts the DVV into motor output signals. Feedback from the muscle spindle modifies the outputs to compensate for perturbations and the result is agonist–antagonist activation signals for both muscle layers. Specifically, the neurocontroller sends control signals (anodic and cathodic currents) for both PPy-DBS layers of the muscle, the anodic current activates one PPy-DBS layer to contract, while the cathodic current induces the other layer to expand, resulting in a coordinated bending movement.

The proposed bio-inspired neurocontroller for voluntary movement control involves connectivity of the motor cortex, parietal lobe, somatosensory areas, globus pallidus, spinal cord, and muscle spindles as follows:

(1) Desired angles ($\theta_{d}$) of the biomimetic muscle are transformed into normalized desired lengths of the muscles virtually ($T_{id}$) as shown in Equations (A1) and (A2). This transformation is sent as reference signals of the *target position vector* (TPV) form (located in area 4) to the cortical neural controller.
(2) In parietal area 5 phasic, a DV of the linear tendon motion (virtually calculated, for this case) in the biomimetic muscle joint is calculated from the comparison of a TPV with a representation of the current position (see Equation (A3)) [42,43,44,45].
(3) The GO signal, an essential component of the basal ganglia nucleus, plays a critical role in representing the *globus pallidus* and actively participating in the regulation of voluntary movement [41,46]. This GO signal is responsible for controlling both the speed of voluntary movement (see Equations (A4)–(A6)) and the level of effort applied in response to obstacles and the speed of reaction to perturbations [47]. Adaptation of the GO signal allows precise regulation of both the onset and velocity of joint movements by adjusting the integration order of the *outflow position vector* (OPV). Furthermore, it ensures synchronous activation and coordination of all muscles in synergy contributing to a harmonized joint movement [47].
(4) The DV, located in area 4, is transformed into a *desired velocity vector* (DVV). To achieve this, a voluntarily adjustable GO signal is introduced, which is multiplied with the DV input to generate the DVV in area 4 associated with phasic movement time (MT). Due to the influence of the adjustable gate signal, the DVV activity (phasic cell activity) is used as a velocity sensitive command (see Equation (A7)) [48,49].
(5) The calculation of the *perceived position vector* (PPV) is carried out in the tonic anterior area 5, as detailed in Equation (A15), where it is obtained by subtracting the spindle-derived feedback of the position error from a referential copy of an OPV from area 4 [50,51].
(6) The OPV activity is in tonic area 4 and obtained by integrating the command DVV with a population of tonic cells in area 4 [49,52]. This tonic cell population plays an essential role in serving as the origin of the efferent copy signal that is utilized in area 5 to calculate PPV. As movement progresses, DV activity in area 5 tends to return to its basal level. This results in the interruption of excitatory input to the phasic cells located in area 4, and ultimately, in the termination of the movement itself, as detailed in Equation (A8).
(7) The existence of a bidirectional connection between the PPV cells in area 5 and the cortical tonic motor cells (OPV) makes it possible for the position command generated in area 4 to follow and adapt to any movement induced by external forces. This bidirectional interconnection plays a crucial role in the prevention of instabilities [53,54].
(8) The *outflow force + position vector* (OFPV) originates from the activity of the cell population associated with both position and phasic-tonic force in area 4. Its function is of great importance, as it facilitates a gradual increase in the force required to counter static and inertial loads (see Equation (A16)). These cells receive inputs from the cerebellum (not modeled in this controller) and from a center that integrates feedback from the neuromuscular spindle. In area 4, phasic–tonic cortico-motoneuronal cells perform a crucial role in allowing the application of the desired force to an obstacle without intervening with precise proprioception (PPV). At the same time, these cells allow TPV to be maintained in case the obstacle yields, as noted in previous studies [55,56].
(9) The *inertial force vector* (IFV) supplies an element that is proportionate to the position error time derivative across the muscle primary spindle signal (Ia) and is identified with phasic reaction time (RT) cell activity in area 4 (see Equations (A11) and (A13)) [57].
(10) The *static force vector* (SFV) plays a key role in providing a proportional element to the integral of the position error. This component is added to the OFPV and controls the load compensation capability in response to disturbances. The identification of this function is carried out in area 4 (see Equations (A12) and (A14)) [47].
(11) Alpha motor neurons (α-MNs) are similar to a controller that supplies action potentials to the extrafusal fibers of skeletal muscles generating muscle contraction forces (see Equation (A17)). They play a fundamental role in generating the desired kinematic output, even under the influence of changing external forces. They integrate the activity of the OFPV and the information provided by type Ia afferent fibers coming from the primary muscle spindles (see Equation (A11)) [47].
(12) The neurocontroller generates its control actions through a dynamic muscle force model (see Equation (A18)) that emulates an elastic tissue with a neuronally adjustable contractile component ($c_{i}$), which confers for agonist–antagonist contractile force ($F_{i}$) development for biomimetic muscle layers. The dynamics of the contractile state ($c_{i}$) of the muscle are achieved by activation of α-MNs, which generate the contractile forces necessary to control voluntary joint movement (see Equation (A19)) [47,58].

The actual angle (θ) of the biomimetic muscle is transformed into virtually normalized tendon lengths ($L_{in}$) as shown in Equations (A9) and (A10). It is used as feedback of the muscle force model and the primary (Ia) and secondary (II) muscle spindles. The control signals generated by the neurocontroller are the agonist–antagonist forces ($F_{i}$) that are transformed into anodic and cathodic electric currents ($T_{EC}$) sent to the corresponding layers of the three-layer artificial muscle (see Equations (A20)−(A22)). In this study, we took into account both positive and negative currents (anodic and cathodic electric currents, respectively) through the WE. These assumptions were translated into a detailed mathematical model, which is presented in Appendix A for precise representation.

This bio-inspired approach allows control of the voluntary movement of the soft biomimetic muscle in a realistic manner based on neurophysiological principles. The controller coordinates the two muscle layers in a manner similar to how the brain orchestrates agonist–antagonist muscle pairs.

**Figure 5:**
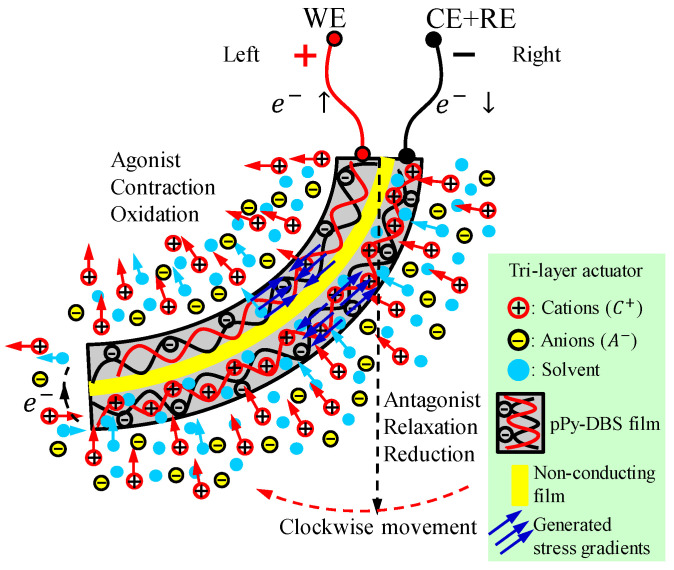
Scheme of the ionic exchanges, clockwise bending movements (CMs), and generated stress gradients during actuation by current flow with anodic polarity (**left**) and by current flow with cathodic polarity (**right**) of the electro-chemo-mechanical triple-layer artificial muscle.

Figure 5 shows the performance of the biomimetic muscle and the cation exchanges and generated stress gradients in each layer. When the action signal is an anodic current flow to the WE, the connected PPy-DBS film (left layer) shrinks by oxidation due to the expulsion of cations and solvent molecules, acts as an agonist, and performs contraction. While the film on the right side connected to the CE + RE receives a cathodic current flow swelling by reduction, causing the influx of cations from the electrolyte, it acts as an antagonist, and performs the relaxation of the actuator. According to Figure 5, the stress gradients are generated due to the electrochemical–mechanical oxidation/reduction reaction of the two PPy-DBS layers of the three-layer actuator, causing clockwise bending movements. This mismatch in the dimensional changes of the two layers attached to the central passive tape induces the bending motion. Specifically, the contracting layer generates compressive stress, while the expanding layer produces tensile stress. The resulting stress gradient across the layer structure generates the bending moment. By changing the direction of electric current flow, the film on the right side acts as the anode, while the film on the left side becomes the cathode, resulting in the generation of anticlockwise bending movements, which are opposite to the previous movements. In this reversal in ion flow, the resulting stress gradients also undergo a corresponding reversal.

## 3. Results

### 3.1. Voltammogram of Triple Layer Biomimetic Muscle

An exhaustive characterization of the biomimetic muscle was performed using the cyclic voltammetry (CV) technique, applying an Ag/AgCl (3 M KCl) electrode as RE, a stainless steel sheet as CE, and short-circuiting the device layers to act as WE. In a solution in water with a LiClO_4_ concentration of 0.1 M, CVs were recorded at room temperature, whose limiting muscle potentials were between −0.805 V and 0.305 V, using a scanning rate of 5 mV/s and to obtain a stationary voltammetric response. The three-layer actuator stabilized after 10 consecutive cycles while remaining in the reversible zone. This stable CV characterized the mechanical electrochemical behavior and the loading capacity of the assembled triple-layer device. 

**Figure 6:**
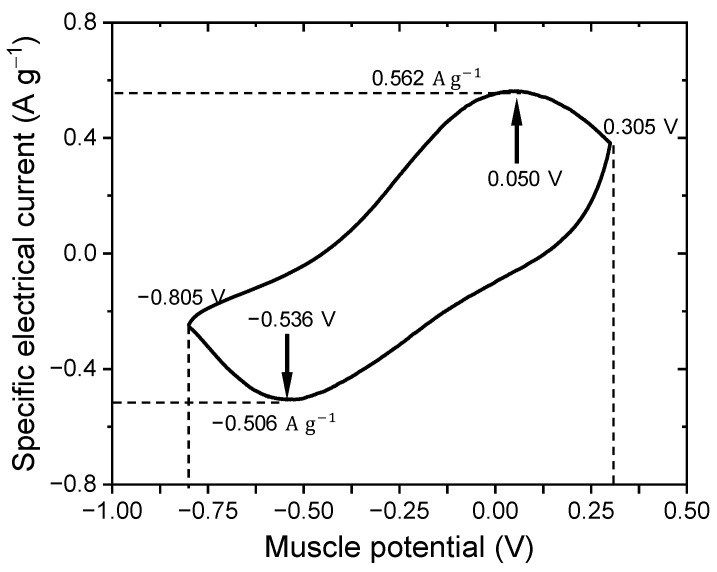
Stationary voltammetric response of biomimetic muscle. After successful completion of 10 consecutive stabilization cycles, the system was subjected to potential cycles ranging from −0.805 V to 0.305 V at a constant scanning rate of 5 mV/s. These cycles were carried out in an aqueous solution with a LiClO_4_ concentration of 0.1 M.

The CV reveals the presence of a cathodic maximum at −0.536 V and an anodic maximum at 0.05 V with specific electric currents of −0.506 A g^−1^ and 0.562 A g^−1^, respectively (see Figure 6). The muscle potential between peaks is 0.586 V and is a result of the slow diffusion of equilibrium counterions through dense PPy-DBS films. The overall shape and peak muscle potentials of the CV in PPy-DBS films are consistent with previous records obtained as mentioned in previous investigations [28,29,30,31,32,33,34]. By integrating the cathodic and anodic currents of the voltammogram, the total charge involved in the process was calculated. In combination with the mass of PPy-DBS films in muscle (m = 6.8 mg), the specific charge involved in the oxidation of the films, referred to as ${Qs}_{ox}$(32.714 C g^−1^), as well as the charge involved in the reduction in the films, known as ${Qs}_{red}$(34.935 C g^−1^), were obtained.

This consequence leads to an outstanding maximum fluctuation in the specific concentration of the cations present in the film, $\left[C^{+}]=Q/(F\cdot m\right)$, where F is the Faraday’s constant (F= 96,485 C mol^−1^) and $\left[C^{+}\right]$ = 3.62 × 10^−4^ mol g^−1^. If during device actuation these magnitudes exceed their maximum value, the actuator abandons its reversible reduction–oxidation behavior and will instead undergo gradual degradation, which is known as overoxidation [6,27]. The stationary voltammogram exhibits a hysteresis loop due to formal potential differences and slow diffusive processes in the PPy-DBS film during sequential reduction and oxidation [59,60,61,62].

The electric potential scan was deliberately interrupted at −0.1 V in the anodic phase of the voltammogram, with the purpose of reaching an intermediate oxidation state. This strategy was designed to enable the subsequent action of oxidizing one film and reducing the other, which would allow for more precise control of voluntary movements.

### 3.2. Sensing and Actuation System of the Biomimetic Device

The muscle actuation and sensing capabilities were studied by immersing the device in an electrolyte solution and applying control signals (anodic/cathodic currents) through a bio-inspired neurocontroller.

Prior to performing the experiments, the biomimetic muscle was brought to the zero position (see Figure 3) for a period of 100 s. The movement control of the actuator is carried out by a bio-inspired cortical neural network. In the sensing characterization, the actuator is subjected to desired position changes of consecutive square waves describing a constant angular movement (±20°, ±30°, and ±40°) at the unrestrained end of the device. The CM represents a change in angular position in the positive direction and the AM is the opposite (see Figure 3 and Figure 5).

**Figure 7:**
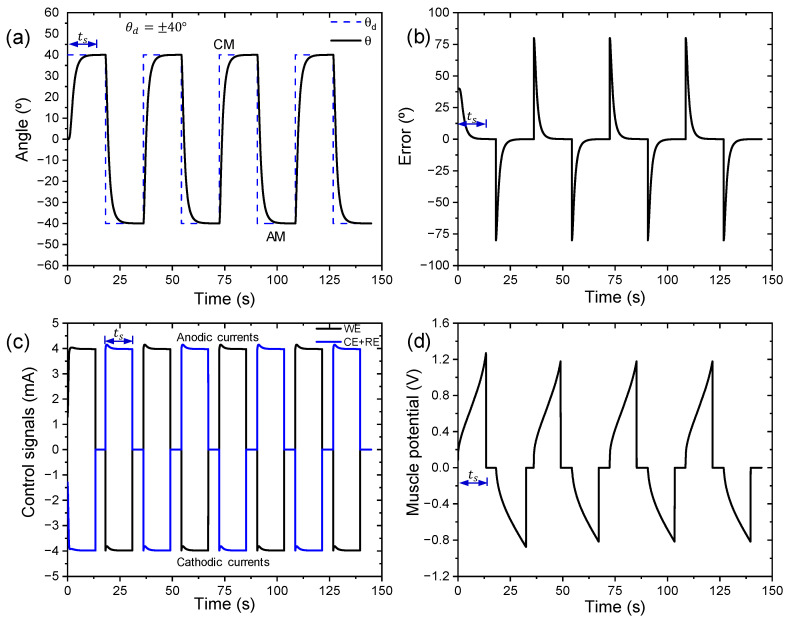
Motion control responses to desired position steps in square waves: (**a**) tracking a square wave path of ±40°; (**b**) tracking error signal; (**c**) anodic and cathodic current control signals from the bio-inspired control algorithm; and (**d**) resulting muscle potential responses. Applying an electric current limit of ±4 mA on the neurocontroller and the system acting in an aqueous solution with a LiClO_4_ concentration of 0.1 M at room temperature (20 ± 1 °C).

Figure 7 shows the movement control of the biomimetic muscle in a sequence of desired position changes (±40°) in the form of square waves. The proposed neurocontroller performs biomimetic muscle motion control with 80° clockwise and anticlockwise motion ranges (see Figure 7a). The settling time ($t_{s}$) for a desired angular position change was 13.333 s consuming a charge of 53.333 mC (Q=It) (see Figure 7a). The tracking error trend to zero was established over the same settling time (see Figure 7b). An electrical current limit ($A_{ec}$) of ±4 mA was set in the neurocontroller (see Figure 7c). The bio-inspired neural control algorithm generates the appropriate control signals (anodic and cathodic currents) for each muscle layer to reach the desired angles and produce continuous movements (CMs and AMs). The neural control algorithm stops sending control signals to the biomimetic muscle when it reaches the desired angular position (see Figure 7c). When a CM occurs, the neural control algorithm sends an anodic current to the layer connected to the WE and a cathodic current to the layer connected to the CE + RE. In the case of an AM, the neural algorithm reverses the roles.

Through the resulting output chronopotentiograms, we see the sensing properties of the biomimetic device with PPy-DBS films when the muscle is subjected to continuous motion control at room temperature in a 0.1 M LiClO_4_ water solution (see Figure 7d).

During the movement of the muscle in Figure 7a, the resulting chronopotentiometric response is shown in Figure 7d. The muscle potential (WE vs. CE + RE) only evolves when the current passes through the WE and stops when the muscle reaches the desired position at the settling time. Positive muscle potentials indicate that the neural control algorithm is sending an anodal current control signal to the muscle layer connected to the WE and producing a CM, while negative muscle potentials indicate that the muscle layer connected to the WE is receiving a cathodic current control signal coming from the neural control algorithm generating an AM (see Figure 7d).

The neurocontroller will perform biomimetic muscle motion control clockwise and anticlockwise in an angular position range of ±20°, ±30°, and ±40°, moving backwards and forwards starting from the vertical position (see Figure 3). During bending motion control of the unrestrained end of the biomimetic muscle and by monitoring muscle potentials, sensing characteristics are extracted as a function of applied currents, temperatures, and different electrolyte concentrations. Sensory analysis of the mentioned variables was carried out using the third cycle of the chronopotentiograms obtained from the desired sequence of device position changes. Angular velocity, charge density, and energy consumption were measured under different conditions to characterize electromechanical performance. A vision tracking system quantified angular displacements during drive cycles.

According to Figure 3 and Figure 7, the angular movement orientation of the three-layer biomimetic muscle indicates that the PPy-DBS film, which acts as the cathode, undergoes an expansion (swells) during reduction, which drives (pushing) the device and promotes the predominant incorporation of cations through an ionic exchange with the solution. Meanwhile, the anode PPy-DBS film contracts during oxidation, as shown in Figure 5, due to the expulsion of cations into solution.

This indicates that the flow of electric current drives redox (reduction–oxidation) processes by facilitating the exchange of ${Li}^{+}$ ions between the electrolyte and the polymer matrix. This exchange of ions, along with solvent molecules (S), e.g., H_2_O, is essential for balancing osmotic pressure. The electrochemical motor reaction involving water exchange is summarized as follows [25,35]:

(1) $${\left[\left(PPy\right){\left({DBS}^{-}\right)}_{n}{\left({Li}^{+}\right)}_{n}{\left(S\right)}_{m}\right]}_{s}⇋{\left[({PPy}^{n+}){\left({DBS}^{-}\right)}_{n}\right]}_{s}+n{\left({Li}^{+}\right)}_{aq}+m(S)+n(e^{-}),$$

where subscripts stand for: s, solid and aq, solution, PPy represents polypyrrole chains, and ${DBS}^{-}$ represents macroscopic anion trapped inside the polymer matrix, and ${Li}^{+}$ represents cations required for charge balance [17,25,35]. The macroscopic volume variation caused by the constant variation in the film composition favors the increase in stress gradients (expansion and compression at the cathode anode, respectively) across the conductive interfaces of the triple-layer biomimetic muscle. Consequently, the unattached end of the biomimetic actuator undergoes continuous angular motion (see Figure 5).

### 3.3. Triple-Layer Biomimetic Muscle as a Current Sensor

The experimental procedure outlined in Figure 7 is used to study the applied current dependence on chronoamperometric responses, with different electrical current limits on the neurocontroller ranging from ±1 to ±10 mA to maintain a desired continuous angular motion at ±20°, ±30°, and ±40°. Experiments were performed at room temperature in an aqueous solution with a LiClO_4_ concentration of 0.1 M. While controlling the triple-layer actuator to describe angular movements mentioned above, muscle potentials were obtained from the chronopotentiograms. 

**Figure 8:**
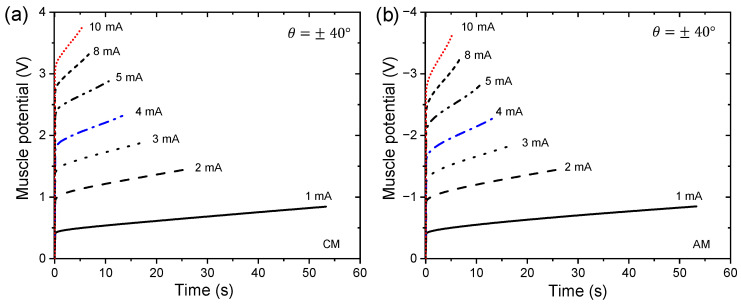
Triple-layer actuator current sensing characteristics: (**a**) chronopotentiograms for clockwise controlled movements (±40°) where the control signals to WE are anodic currents and (**b**) for anticlockwise controlled movements where WE receives cathodic currents from the neurocontroller (electrolyte: an aqueous solution with a LiClO_4_ concentration of 0.1 M at room temperature).

Figure 8 shows the third chronopotentiograms of movement control in a clockwise and anticlockwise direction stabilized for an angular displacement of ±40° with different limits of currents in the neurocontroller. The neurocontroller sends the control signals (anodic and cathodic currents) to the triple layer muscle, where we observe that higher currents induce muscle potentials (potential difference between the PPy-DBS films in the device) more at the starting point of the movement; this is caused by various types of resistance associated with the PPy-DBS film device (e.g., the reaction resistance, the resistance to counterion diffusion, resistance to aqueous solution, or resistance to charge transfer). After this first step, there is a gradual increase in muscle potential.

This evolution takes place at higher potentials when clockwise movements are performed and the WE receives increasing anodic currents sent by the neurocontroller, following the electrochemical reaction of the PPy-DBS film (see Figure 8a). In anticlockwise movements where the neurocontroller sends increasing cathodic currents to the WE, the muscle potential can be observed to gradually decrease towards higher negative potentials (see Figure 8a).

Any chemical or physical variable that affects the rate of a reaction (oxidation or reduction) should induce current-induced changes in muscle potential. The evolution of muscle potential and the current flowing over a specific period characterize the consumed electric energy by electric devices ($E_{C}=Q\;E$). This suggests that when the biomimetic muscle is working, it detects any variable (such as temperature, concentration, pressure, presence of obstacles, or mechanical stress) modifying muscle potential and adjusting to the required electrical energy. During movement control, the neurocontroller sends the necessary control signals (anodic or cathodic current flows) to the triple-layered muscle, and the specific electrical energy used to describe angular controlled motions of ±20°, ±30°, and ±40° is calculated as $E_{s}=(I/m)\int Edt$, where E represents the potential; t, the time; and I, the applied electric current. The integral, ∫Edt, represents the area under test chronopotentiograms.

**Figure 9:**
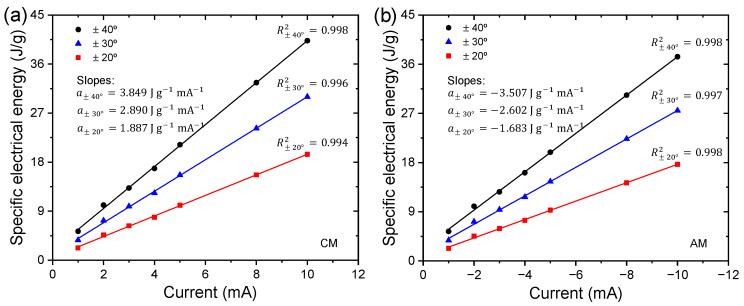
Linear relationship between specific electrical energy consumption and different control signals applied to the WE for (**a**) CMs (applying anodic currents) and (**b**) AMs (applying cathodic currents) of controlled angles (±20°, ±30°, and ±40°). R^2^ is the linear regression coefficient. The weight of PPy-DBS films on the device was 6.8 mg.

Integrating Figure 8a,b, we obtain the specific electrical energy consumed by the device when working with different electrical currents for controlled movements of ±40° during the current sensing process applied to the trilayer device. Figure 9 reveals that the specific electrical energy consumption for describing rotational motions (±20°, ±30°, and ±40°) is a linear function of the control signal (applied current). Three linear relationships are established between specific electrical energy consumption, one for each angle considered, as a function of applied currents. Similar changes in energy evolution were observed during clockwise and anticlockwise movements. The slopes ($Jg^{-1}{mA}^{-1}$) indicate the increase in specific electrical energy consumed as the magnitude of the applied current increases by one mA, quantifying the electrical current sensing capability of this specific actuator device. Slopes increase as the desired angles consistently increase: as the applied current period extends, the reduction or oxidation reactions exhibit higher resistance in the most reduced or oxidized states of the conductive polymer, in this case, the PPy-DBS films.

When a CM control is performed, the WE receives a control signal (anode current) from the neurocontroller, observing that there is a linear relationship of the applied current with respect to the specific electrical energy consumed (see Figure 9a), and the same happens when an AM control is performed where the WE receives cathodic currents (see Figure 9b). These results emphasize that the trilayer device can sense any variation in electric current during operation. This device, which connects with only two cables (see Figure 3), is capable of handling both activation signals (control signals) and detection signals (potential). The trilayer device represents a biomimetic muscle as well as sensor actuator.

### 3.4. Triple-Layer Biomimetic Muscle as a Temperature Sensor

According to the experimental technique illustrated in Figure 7, the influence of temperature on the chronopotentiometric responses, in a range from 5 to 35 °C, was studied when subjected to desired position changes of consequential square waves describing a constant angular motion (±20°, ±30°, and ±40°) at the unrestricted end of the trilayer device. An electrical current limit ($A_{ec}$) of ±4 mA was set on the neurocontroller, and the experiments were carried out in a 0.1 M LiClO_4_ aqueous solution.

**Figure 10:**
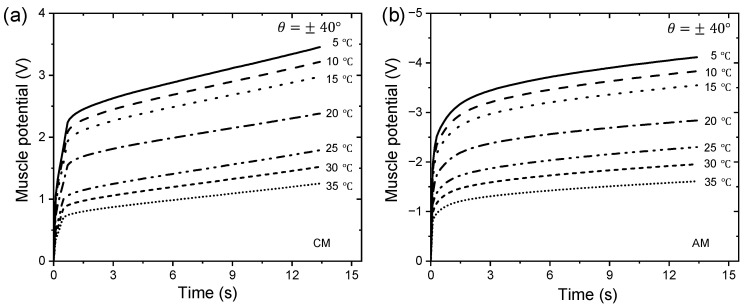
Muscle potential for temperature sensing properties of the trilayer device: chronopotentiograms with third cycle data (**a**) for controlled CMs (±40°) where the WE receives control signals (anodic currents) and (**b**) for controlled AMs where the WE receives cathodic currents from the neurocontroller with $A_{ec}$ = ±4 mA (electrolyte: an aqueous solution with a LiClO_4_ concentration of 0.1 M).

Figure 10 shows the muscle potential responses for a desired constant angular movement of ±40° (third cycle chronopotentiogram data), and where the neurocontroller controls clockwise and anticlockwise movements by sending anodic and cathodic current control signals to the WE, respectively. At lower temperatures, the muscle potential is higher due to the resistivity associated with the PPy-DBS material, and as the temperature increases, the resistivity of the material decreases along with the muscle potential.

In both CMs and AMs, the anodic and cathodic currents flowed for a time period ($t_{s}$) of 13.333 s for ±40°, 10 s for ±30°, and 6.666 s for ±20° with a consumption of ±53.333 mC, ±40 mC, and ±26.666 mC, respectively, in each direction. Whatever the angle considered, the muscle potential decreases at higher experimental temperatures, which means that a small increase in the temperature of the reaction will produce a marked increase in the magnitude of the reaction rate constant according to the Arrhenius equation.

**Figure 11:**
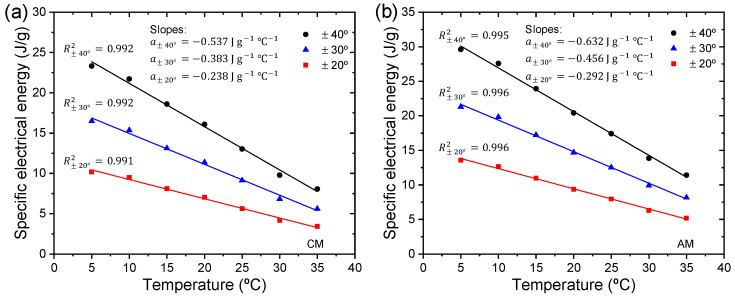
Linear relationship of the specific electrical energy consumed at different temperatures corresponding to the experimental results for controlled movements (±20°, ±30°, and ±40°): (**a**) for controlled CMs where the WE receives anodic currents from the neurocontroller and (**b**) for controlled AMs where the WE receives cathodic currents. The electrical current limit established for the neurocontroller was $A_{ec}$ = ±4 mA. R^2^ is the linear regression coefficient. The weight of PPy-DBS films on the device was 6.8 mg.

By integrating Figure 10a,b, the specific electrical energy consumed by the trilayer device was calculated at various temperatures for controlled ±40° movements. In Figure 11, a linear decrease in specific electrical energy consumed is seen as temperature increases, while constant angular displacements (±20°, ±30°, and ±40°) are described.

Whatever the angle considered, the slopes (J g^−1^ °C^−1^) formed when CMs and AMs are performed represent the increase in specific electrical energy consumed when the temperature decreases by one degree Celsius. Figure 11 shows an increase in slopes as desired angles increase in magnitude. These slopes quantify the actuator device’s capability of sensing temperature changes. Figure 11 indicates that our triple-layer device can detect temperature changes during operation.

### 3.5. Triple-Layer Biomimetic Muscle as Electrolyte Concentration Sensor

Studies were conducted on the chronopotentiometric responses in aqueous solutions containing LiClO_4_ at room temperature, varying electrolyte concentrations in a range of 0.01 to 1 M, while the neurocontroller controls the unrestricted end of the trilayer device to desired position changes through consequential square waves describing constant angular movements of ±20°, ±30°, and ±40°. In the experiments, an electric current limit ($A_{ec}$) of ±4 mA was set on the neurocontroller. Electrolyte concentration and muscle potential depend on the same oxidation/reduction states corresponding to angular movements of ±20°, ±30°, and ±40° reached at $t_{s1}$ = 6.666 s, $t_{s2}$ = 10 s, and $t_{s3}$ = 13.333 s, respectively.

**Figure 12:**
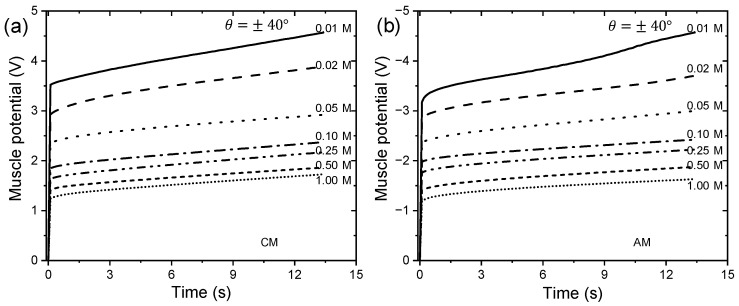
Muscle potential of the triple-layer actuator for electrolyte concentration sensing characteristics: chronopotentiograms with third cycle data (**a**) for controlled CMs (±40°) where the WE receives control signals (anodic currents) and (**b**) for controlled AMs where the WE receives cathodic currents from the neurocontroller with $A_{ec}$ = ±4 mA (electrolyte: LiClO_4_ aqueous solution at room temperature).

From the electrochemical motor reaction (Equation (1)), it can be deduced that increasing electrolyte concentrations facilitate the reaction’s control by CMs and AMs of the triple-layer muscle, resulting in lower muscle potentials, as observed in the results shown in Figure 12 for a desired constant angular motion of ±40° (data from the third cycle chronopotentiogram). According to Figure 12, at a lower electrolyte concentration, the muscle potential is higher due to the resistivity associated with the PPy-DBS material, and as the electrolyte concentration increases, the material’s resistivity decreases, along with the muscle potential.

**Figure 13:**
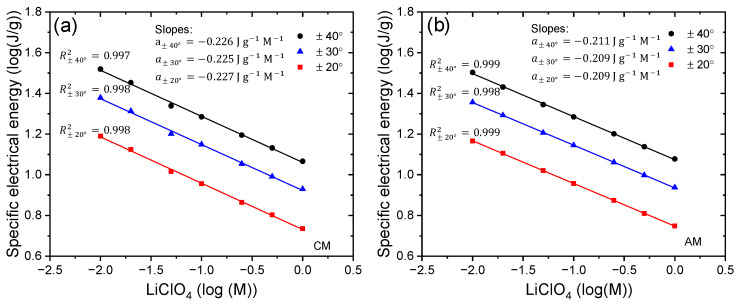
Linear relationship of the specific electrical energy consumed at different electrolyte concentrations corresponding to experimental results for controlled movements (±20°, ±30°, and ±40°): (**a**) for controlled CMs where the WE receives anodic currents from the neurocontroller and (**b**) for controlled AMs where the WE receives cathodic currents. The electrical current limit established for the neurocontroller was $A_{ec}$ = ±4 mA. R^2^ is the linear regression coefficient. The weight of PPy-DBS films on the device was 6.8 mg.

By integrating the experimental curves of the chronopotentiograms obtained from the constant controlled angular movements of ±20°, ±30°, and ±40° (see Figure 12), we obtain Figure 13, which shows that to describe a constant controlled angle, the specific electrical energy consumed, under the flow of a control signal (anodic or cathodic current) sent to the WE by the neurocontroller during a settling time, increases with decreasing electrolyte concentration.

Figure 13 shows the linear relationship between electrolyte concentrations and specific electrical energy during constant angular controlled movements, demonstrating that the trilayer device functions as a sensor of electrolyte concentrations while working.

The reduction (AM) and oxidation (CM) slopes (J g^−1^ M^−1^) indicate the increase in specific electrical energy consumed per unit change in electrolyte concentration, quantifying the electrolyte concentration sensing capability of this actuating device.

### 3.6. Muscular Angular Rate versus the Driving Current

The influence of electric current density on angular velocity and charge density was studied over a range of ±0.375 to ±7.5 mA∙cm^−2^ when consecutive square waves describing a constant angle of movement of ±45° (±π/4 rad) at the unrestricted end of the trilayer device were subjected to desired position changes.

**Figure 14:**
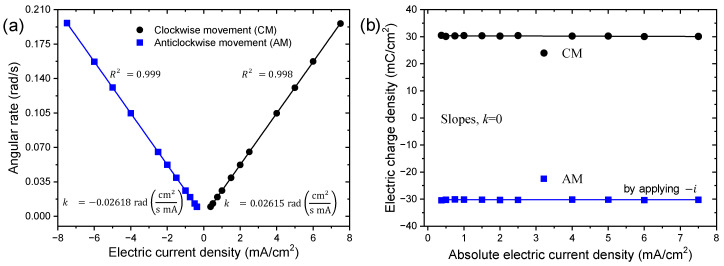
Results of experiments on the influence of angular velocity and charge density: (**a**) linear relationship between angular velocity and applied current density determined from the time required to perform an angular movement of ±45° under the flow of various current densities sent from the neurocontroller of ±0.375 to ±7.5 mA∙cm^−2^, for the trilayer device; (**b**) linear relationship between various experimental current densities and charge density consumed by the trilayer device for ±45° displacement (electrolyte: an aqueous solution with a LiClO_4_ concentration of 0.1 M at room temperature). R^2^ is the linear regression coefficient.

Figure 14 shows the time needed and measured by the unrestricted end of the trilayer device to describe an angle of ±45° (beginning from upright position), controlled by the neurocontroller through the control signals applying a constant current density flow.

As shown in Figure 14a, the angular velocity (*ω*) is represented as a linear relationship of the applied current density and is characterized by:

(2) ω=k·J,

where *ω* is the angular velocity in rad/s, the slope, k, is a constant value and is the described angle of a PPy-DBS surface of one square centimeter per unit time and per unit applied current in rad cm^2^ s^−1^ mA^−1^, and J is the electric current density in mA cm^−2^.

The slope in Figure 14a, k was 26.1 × 10^−3^ rad cm^2^ s^−1^ mA^−1^, noting that the higher the angular velocity, the higher the electric current density in both CMs and AMs. Equation (2) is shown as a characteristic magnitude for artificial muscle control. This equation asserts that the electrochemical muscle functions as an electric device, i.e., the current density intensity controls the rotational motion velocity. Therefore, the motion is accelerated by increasing the current density and slowed down by decreasing the applied current density. The movement stops when the desired angle is reached and the neurocontroller switches off the current density flow. Under the control of the neurocontroller, a phenomenon previously observed in PPy-DBS/tape [31,32,33] or PPy-ClO_4_/tape devices [15,25], the direction of movement is inverted by changing the direction of current density flow to WE.

Regardless of the experimental electrical current used, the electric charge density consumed, *φ* (whether anodic or cathodic) to achieve an angular displacement of ±45° (±π/4 rad), remains constant (±30 mC cm^−2^) at any current density (see Figure 14b).

### 3.7. Consumed Electrical Charge Density and Angular Position

The relationship of angular position and electric charge density was studied for different applied charge densities ranging from 0.5 to 6 mA∙cm^−2^ when subjected to changes in different desired angular positions between 0° and 45° and describing constant angular movements at the unrestricted end of the trilayer device.

**Figure 15:**
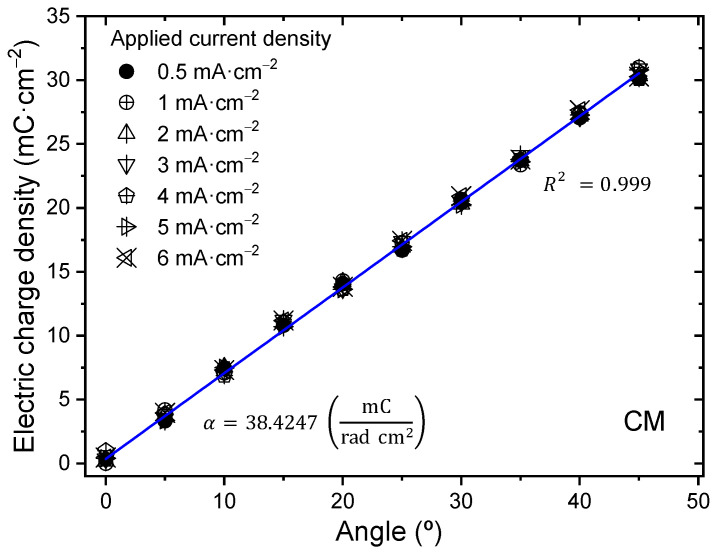
Linear relationship between angular position and consumed electrical charge density by the triple layer device describing different angles (between 0° and 45°) with different anodic current densities sent by the neurocontroller describing CMs (electrolyte: an aqueous solution with a LiClO_4_ concentration of 0.1 M at room temperature). R^2^ is the linear regression coefficient.

In Figure 15, electrical charge densities are consumed to describe different angles (0° to 45°) with CMs, while the neurocontroller applies control signals of different current densities (0.5 to 6 mA∙cm^−2^). The results confirm that the same electric charge density is consumed to produce a bending movement with the same angle, regardless of the current density used in the test. The characteristic magnitude is the slope, α, which has a value of 38.4247 mC∙cm^−2^∙rad^−1^.

The required electric charge density (φ) to reach any new angular position (θ) from the current position is determined by the following calculation:

(3) φ=α·θ.

Therefore, an electrochemical positioning device can be conceptualized as an artificial muscle. The constant, α, is the electrical charge density consumed to represent a continuous angle of one radian. From Equations (2) and (3), it can be inferred that α=1/k, a relationship supported by experimental data while considering the inherent margin of error in measurements. 

## 4. Discussion

The developed triple-layer biomimetic muscle demonstrates promising capabilities as an artificial muscle technology. The integrated sensing and actuation properties allow these soft actuators to closely mimic natural muscle function. 

Additionally, this device replicates several key properties and functions of natural muscle tissue: dual actuation and self-sensing capabilities (the muscle can simultaneously contract/expand and transduce physical/chemical signals from the environment); electrochemical stimulation (the muscle is driven by electrochemical reactions and ion fluxes, akin to neural activation of muscle fibers); biomimetic flexibility (the soft, organic conducting polymer layers enable lifelike bending motions); sensory feedback (monitoring muscle potential provides real-time proprioceptive feedback for closed-loop control, much like natural stretch receptors); agonist–antagonist coordination (independent control of the two muscle layers allows biomimetic activation of opposing muscle pairs); voluntary movement (the bio-inspired neurocontroller elicits dexterous and robust motion control, replicating corticospinal connections); dynamic adaptability (the muscle adjusts to forces and perturbations using physiological force/length/velocity relationships); and multifunctionality (integrated sensing, actuation, and control support versatile capabilities in a single soft material system). There are several key findings and implications that merit further discussion.

### 4.1. Actuation Mechanism and Performance

Electromechanical transduction in conductive polymeric PPy-DBS films allows reversible bending movements under applied control signals (anodic and cathodic currents) through a neurocontroller. This action is driven by ion exchange between the electrolyte and the polymer, which induces swelling and contraction of the PPy-DBS layers. The resulting stress gradients across the multilayer structure drive to biomimetic bending movements.

The maximum specific charge ($Q_{ms}$) exchanged during redox cycling determines that if during device actuation this magnitude exceeds its maximum value, the artifact loses its reversible redox behavior and will suffer a progressive degradation, reaching overoxidation [6]. Cyclic voltammetry results indicate a maximum specific charge capacity of $Q_{ms}$ = 35.993 C g^−1^, which is consistent with previous PPy-DBS films [26,27,32,33]. This loading capacity is related to the changes in oxidation/reduction state and volume change response that drive performance [35].

The trilayer actuator with one of the PPy-DBS films acting as the cathode (CE + RE) and the second as the anode (WE) under a control signal (anodic or cathodic current) applied constant per unit time acts as a flexible battery with a charge/discharge of 7.842 C g^−1^ to achieve a continuous angular motion of ±40° or 17.647 C g^−1^ for ±90°. Our work conditions remained stable as we operated far from the limit capacity of PPy-DBS films (35,993 C g^−1^), i.e., we avoided degradation conditions.

**Figure 16:**
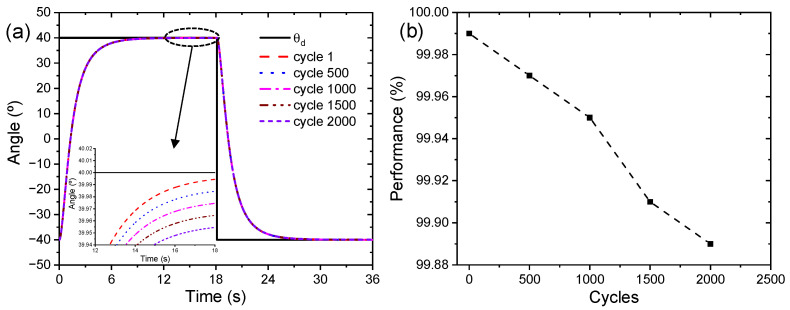
Stability test: (**a**) motion control responses to desired square wave position steps (±40°) at frequency 27.7 mHz (2000 cycles); (**b**) triple-layer device performance vs. number of cycles. Applying an electric current limit of ±4 mA on the neurocontroller and the system acting in an aqueous solution with a LiClO_4_ concentration of 0.1 M at room temperature (20 ± 1 °C).

The suitability of this triple-layer device in artificial muscles, soft robotics, and multifunctional devices requires verification of the long-term stability of the actuation response. Through angular displacement metric monitoring and long-term testing, we analyzed the device performance. Figure 16 presents the performance of the triple-layer biomimetic device for 2000 actuation cycles, where the efficiency decays to 0.11%. The error after 2000 actuation cycles was 0.046°, indicating that the device is stable in the long term.

In [59], they studied the lifetime of conductive polymer films, especially PPy-DBS, establishing practical levels of 10,000 cycles, although other PPy-DBS films may last longer in actuation cycles. Additionally, they concluded that, even if all process parameters and conditions are the same, there is great uncertainty as to the lifetime of these films before failure. Harjo et al. [60] show that the PPy-DBS film exceeds 10,000 cycles of actuation where they reveal that a small creep appears in the conductive film. Khuyen et al. [61] confirm that finding with a value of 0.34%. Creep is essentially a displacement from the initial position, which is partly attributed to irreversible loading [17]. In previous research, studies were conducted to analyze creep under high loads during reversible redox cycling in linear PPy actuators [62].

The response time of this actuator is slower than that of muscle tissue, on the order of seconds rather than milliseconds. This is probably due to the relatively slow ion diffusion within the thick polymer films. Reducing the thickness of the nonconductive film or using electrolytes with higher diffusivity could potentially improve response times.

Overall, while performance does not yet match natural muscle, the actuation stresses and biomimetic flexibility represent excellent progress for soft actuators. Ongoing research into composition, microstructure, ion transport, and device engineering may help achieve improvements in speed, strength, efficiency, and durability.

### 4.2. Integrated Sensing Capabilities

Fundamentally, the muscle potential varies sensitively with applied current, temperature, and ionic concentration during actuation cycles. This demonstrates the ability of conductive polymeric films (PPy-DBS) to simultaneously transduce physical and chemical signals from the local environment into electrical signals, analogous to biological mechanoreceptors and chemoreceptors.

Chronopotentiometric measurements under varying control signals (electrical currents) show sensitive responses in muscle potential, indicating that muscle potential increases proportionally with the driving electrical current sent through the neurocontroller. This is in line with theoretical models relating electrode potential to reaction kinetics and charge transfer. Muscle potential increased linearly with increasing amplitude of the applied control current during actuation cycles. This demonstrates the ability to sense the driving electrical current through changes in muscle potential. The consumed specific electrical energy to represent angular displacements performing CMs and AMs is a linear function of the applied control signal (anodic or cathodic current) in the triple-layer actuator. Additionally, the triple-layer actuator exhibits more self-sensing sensitivity to variations of applied currents in actuation for larger ranges of controlled bending motion (see Figure 9).

Muscle potential varies inversely with temperature, indicating that as temperature increases, muscle potential decreases, following theoretical expectations due to the exponential dependence of the reaction rate constants. The muscle’s specific electrical energy consumption for controlled angular movements decreases linearly as the temperature increases. In addition, when the range of controlled bending motion is wider, the actuator becomes more sensitive to temperature variations (see Figure 11).

The concentration dependence is more complex and is related to ion partitioning and diffusion effects, but still shows systematic variation. Muscle potential decreased exponentially as the electrolyte (LiClO_4_) concentration was increased from 0.01 to 1 M. Electrolyte concentration and muscle potential profile demonstrate that the device has the capability to sense changes in ionic composition of the surrounding fluid environment. There is a linear relationship between variations in electrolyte concentrations and the consumed specific electrical energy during constant angular controlled motions. This consumed energy decreases as the electrolyte concentration increases. Furthermore, the triple-layer actuator exhibits the same sensitivity of the self-sensing of electrolyte concentrations at any range of controlled bending motion (see Figure 13).

In this study, we found linear relationships between muscle potential and temperature/current, and an exponential relationship between potential and electrolyte concentration. All this demonstrates that the triple-layer actuator functions as a sensor of electrolyte concentrations, temperatures, and currents while working.

In the study of the influence of electric current density with angular velocity, a linear relationship between electric current density and angular velocity was determined, indicating that as electric current density increases, angular velocity also increases. In the same way, a linear dependence was found between the electric current density and the consumed electric charge density, showing that the consumed charge density for a certain controlled angle does not vary at any applied current density. Additionally, a linear relationship was found between variations of desired angular positions and the consumed electric charge density per angle at different applied current densities, indicating that as the desired angle increases, the consumed electric charge density for that angle increases without variation at any applied current density. In summary, the current density controls the speed of motion but not the total charge consumed per angle. This allows for modeling angular velocity, charge density, and desired angle independently as a function of current density. These insights further reveal the integrated electro-chemo-mechanical transduction in the biomimetic muscle system.

This integrated sensing provides real-time feedback for closed-loop control of actuator positions and responses to perturbations. Monitoring of muscle potentials during operation can enable self-regulated actuation similar to natural muscle spindles and Golgi tendon organs. The chronopotentiometric technique provides a simple yet powerful method to exploit the sensing capabilities. Further signal processing and multivariable analysis could elucidate complex relationships between the muscle potential and environmental conditions. Overall, this dual functionality allows the conducting polymer muscle to emulate the sensing actuation behavior of natural muscle tissues.

The soft biomimetic actuator presented in this paper relies on cation exchange with the surrounding electrolyte solution to drive actuation and self-sensing. While we used aqueous LiClO4 solutions in our experiments, the actuator can also operate with other aqueous electrolyte solutions that permit cation exchange, such as NaCl, RbCl, KCl, CsCl, MgCl_2_, CaCl_2_, and NaClO_4_ salts, among others [23,24,25,26,27,60,61]. Cations can effectively intercalate in and out of the PPy-DBS layers during redox reactions. Anions have a lesser impact on the performance of this type of material as fabricated in this paper. Other researchers worked with conducting polymers (monolayers or bilayers) that exchange anions and demonstrated the possibility of using various electrolyte solutions allowing this ion exchange with the polymer [16,17,25,27,32,33,34,35,36,37]. They observed that the bending movement depends on the specific electrolyte solution used and the electrochemomechanical reaction, resulting in different muscle potentials with varying slopes for each of the salts studied. We hypothesize that the trilayer biomimetic muscle, when immersed in different aqueous electrolyte solutions with various cations, can sustain actuation and self-sensing, effectively functioning as electrochemomechanical motors [27,60,61].

The limitations of self-sensing or actuation in artificial conductive polymer actuators may be due to: (a) ionic concentration, as there are optimal concentration ranges for conductivity and osmotic pressure effects depending on the material manufactured (too diluted or concentrated can limit actuator performance [60]); (b) viscosity or the dielectric constant of the solvent affect the ion flows that influence actuation (water is ideal, but some organic solvents may also work); and (c) the electrolyte must allow for a sufficient voltage range to oxidize and reduce the polymer layers for actuation [61]. In medical applications, it is preferable to use biocompatible salts, such as sodium chloride, to maximize safety. There are some general guidelines: salts with small cations in aqueous solutions at low concentrations work best [32,34,61]. Solutions that alter electrochemical reactions, polymer morphology, ion and solvent transport, or biocompatibility will impair performance. The compatibility of new chemical electrolytes should be evaluated. In general, we can assume that actuators with PPy-DBS films will work with a range of electrolyte solutions with suitable properties and concentrations. However, extreme conditions may limit their applicability. 

According to the experimental results presented in Figure 9, Figure 11, and Figure 13, we propose a linear multi-variable model representing the self-sensing capabilities, characterized by

(4) $$E_{s}={(\beta }_{1}\theta +C_{1})\chi +{(\beta }_{2}\theta +C_{2}),$$

where χ represents the objective variables of current (i) in mA, temperature (T) in °C, and electrolyte concentration in log(M); θ is angular displacement amplitude in rad; $\beta_{i}$ are dependent constants; and $C_{i}$ is a constant. 

For variations of currents (χ=i) when working with CMs, the constants acquire the following values: $\beta_{1}=2.8105$ J g^−1^ mA^−1^ rad^−1^, $\beta_{2}=1.7527$ J g^−1^ rad^−1^, $C_{1}=-0.067$ J g^−1^ mA^−1^, and $C_{2}=-0.6872$ J g^−1^; For AMs, $\beta_{1}=-2.6126$ J g^−1^ mA^−1^ rad^−1^, $\beta_{2}=2.0735$ J g^−1^ rad^−1^, $C_{1}=0.1377$ J g^−1^ mA^−1^, and $C_{2}=-0.6199$ J g^−1^. For temperature variations (χ=T) when working with CMs, the constants are: $\beta_{1}=-0.4275$ J g^−1^ °C^−1^ rad^−1^, $\beta_{2}=21.368$ J g^−1^ rad^−1^, $C_{1}=0.0613$ J g^−1^ °C^−1^, and $C_{2}=-3.3884$ J g^−1^; For AMs, $\beta_{1}=-0.4879$ J g^−1^ °C^−1^ rad^−1^, $\beta_{2}=25.763$ J g^−1^ rad^−1^, $C_{1}=0.0505$ J g^−1^ °C^−1^, and $C_{2}=-2.8191$ J g^−1^. For variations of electrolyte concentrations (χ=log(M)) when working with CMs, the constants acquire the following values: $\beta_{1}≅0.002$ log(J g^−1^) log(M)^−1^ rad^−1^, $\beta_{2}=0.4729$ log(J g^−1^) rad^−1^, $C_{1}=-0.2285$ log(J g^−1^) log(M)^−1^, and $C_{2}=0.4097$ log(J g^−1^); for AMs, $\beta_{1}≅-0.0023$ log(J g^−1^) log(M)^−1^ rad^−1^, $\beta_{2}=0.4682$ log(J g^−1^) rad^−1^, $C_{1}=-0.2079$ log(J g^−1^) log(M)^−1^, and $C_{2}=0.4288$ log(J g^−1^). The constants were obtained from the graphs mentioned above.

Equation (4) operates with the physical variables from the experiments. In the model, the second term of the equation determines the cut-off point of the linear relationship, depending on the amplitude of the angular actuation displacement, while the first term of the equation represents the slope of the resulting line. When there are variations in electrolyte concentrations, the constant $\beta_{1}$ is approximately zero, indicating that the slope formed does not depend on the angular displacement, but the cut-off point of the linear relationship is not.

Other researchers modeled the evolution of the consumed specific electrical energy, $E_{s}$, under the flow of a constant current [6,25,26,32,34,37]. Indicating that the energy consumed by the reaction driving the actuation of the PPy device must adapt instantaneously to mechanical (entrained masses), thermal, chemical, or electrical working conditions [25]. In addition, the self-multisensory/actuator equations describe the physical and chemical understanding of the reaction energy of self-multisensory electrochemical motors [6].

### 4.3. Voluntary Movement Control

The integrated bio-inspired neural control system exhibits several key features that mimic biological motion regulation: agonist–antagonist coordination (the controller activates opposing muscle layers similar to natural muscle pairs to produce coordinated motions); sensory feedback (signals from virtual muscle spindles allow compensating for disturbances, resembling proprioceptive regulation); adaptive force control (static and inertial force commands adapt to loads, much like the response of real muscles); voluntary movement (the system converts high-level position goals into biomimetic muscle activations for dexterous control); variable speed control (the globus pallidus-based gating element modulates velocity and effort, comparable to motor pathways); dynamic stability (bidirectional connectivity prevents instabilities, mirroring intracortical connections in the brain); incremental trajectory generation (smooth trajectories are produced by interpolating between initial and final states, as in the CNS); threshold-linear tension (the muscle model exhibits a biologically realistic tension length curve adjustable by neural activation); and physiological control principles (the system incorporates features such as length/velocity/force relationships and muscle synergies).

Experiments with the control voluntary bending movement allowed for systematically characterizing the actuation, and self-sensing of the triple-layer soft muscle system in a series of test conditions. The bio-inspired neural control system also provides an advanced strategy for lifelike motion control. The corticospinal model coordinates agonist–antagonist activation similar to the motor pathways in the human nervous system. This achieves accurate and smooth trajectory tracking for arbitrary joint movements. 

The dynamic muscle model relating input currents to muscle forces and positions also adds a level of biomimicry. The threshold-linear tension curve with neurally adjustable offset resembles biological muscle function. This allows for modulating the effective rest length to control contraction. The proportional–integral control of static and inertial loads also parallels the responses of mono- and bi-articular limb muscles. This compensates disturbances to enable dexterous and robust motion control even under external forces.

This neural control demonstrates that conducting polymer actuators can be precisely maneuvered, similar to skeletal muscles in biological organisms. The voluntary motion control and dynamic biomimetic response represent a major advance over basic open-loop driving of artificial muscle devices.

Nonetheless, enhancements in the control algorithms could improve the response accuracy and speed. Incorporating sensory feedback terms and optimal control methods such as model predictive control could enhance the trajectory tracking. Learning-based approaches such as reinforcement learning may also provide adaptive and flexible control.

The current model focuses on 2D single-joint movements for simplicity. Expanding the control framework to multi-joint 3D movements could further demonstrate the capabilities. Overall, the voluntary motion control provides an excellent foundation to develop advanced artificial muscle control systems.

### 4.4. Applications and Future work

This biomimetic muscle holds promise for diverse applications in soft robotics, prosthetics, wearable devices, and biomedical systems. The integrated actuation and sensing enable multi-functional capabilities well suited for human–machine interfaces.

For instance, soft actuators based on this technology could mimic natural musculoskeletal structures for compliant, adaptable robots. The sensing capabilities would allow proprioceptive feedback for delicate environmental interactions.

The conducting polymer actuators may also find use in powered prosthetic limbs or exoskeletons, providing biomimetic actuation with sensory information on limb/joint angles and loads. This could restore more natural motor control and feel for amputees or paralysis patients. 

Wearable applications such as active compression garments and assistive suits could likewise benefit from the muscle-like actuation with sensing. Potential biomedical uses range from steerable catheters to artificial sphincters to biomechatronic tissues.

Future work should focus on improving the performance to reach natural muscle capabilities, enhancing the sensory transduction and closed-loop control, and demonstrating applications in biomimetic systems. Additional directions include electrode patterning for complex deformation modes, hybrid composites with grafted polymer brushes to emulate the extracellular matrix, and tandem configurations to boost force capabilities. Exploring biocompatibility could also enable medical implants and interfacing.

This triple-layer biomimetic muscle exhibits excellent progress towards replicating muscle function with soft, electroactive materials. The integrated self-sensing and actuation closely mimics natural behavior. The voluntary motion control highlights the potential for dexterous, robust control. Ongoing research can build on these results to further advance artificial muscle systems with lifelike performance and multi-functional capabilities. The technology holds great promise for next-generation soft robotics, human–machine interfaces, wearable devices, and biomedical technologies.

## 5. Conclusions

This paper introduces the integration of a bio-inspired neurocontroller for controlling desired angular movements of a triple-layer biomimetic muscle, analogous to biological muscles. The triple-layer biomimetic muscle actuator presented in this work demonstrates both sensing and actuation capabilities. The conducting polymer-based soft actuator can detect changes in current, temperature, and concentration, while also undergoing controlled motion. This replicates key multifunctional properties of natural muscles, which can sense variables such as length and force during motion regulation. The integrated cortico-spinal neural network controller can successfully produce smooth and stable voluntary movements of the muscle actuator’s free end to desired angles of ±20°, ±30°, and ±40°. This neurobiologically inspired control scheme provides an effective interface between the soft biomimetic muscle and high-level voluntary movement commands.

Monitoring the muscle potential during actuation reveals a linear relationship between potential and the amplitude of applied current. This enables the muscle to precisely quantify the driving current applied to it, allowing it to function as a current sensor for bio-feedback control. Experiments also demonstrate that the muscle potential exhibits a linear dependence on temperature, permitting the muscle to sense environmental temperature changes. Varying the electrolyte concentration induces exponential changes in the muscle potential response, providing concentration sensing abilities. All this demonstrates that the triple-layer actuator functions as a sensor of electrolyte concentrations, temperatures, and currents while working. The triple-layer muscle presents a linear dependence between electric current density and angular velocity, as well as with the electric charge density consumed per angle. It also shows a linear relationship between variations of desired angular positions and the electric charge density consumed per angle at different applied current densities.

The integrated actuation and sensing features of this biomimetic muscle represent a significant advance for soft robotics, prosthetics, and other biomedical devices requiring biomimetic functionality. This multi-functional conducting polymer muscle overcomes limitations of previous artificial muscle designs lacking either integrated sensing or precise control. Overall, the simultaneous actuation and sensing capabilities prove that this novel muscle actuator represents an important milestone in developing biomimetic soft robots and biomedical devices.

## Data Availability

Data are contained within the article.

## Appendix A

According to Figure 4, the proposed cortical network for motion control is governed by the following system of equations. The desired position of the artificial muscle is transformed and normalized ($T_{d}$) to muscle lengths with values from 0 to 1, where 1 represents the maximum contraction state of the trilayer muscle and 0 represents maximum relaxation state, as follows:

(A1) $$T_{1d}=\frac{\theta_{d}+\pi /2}{\pi },$$

(A2) $$T_{2d}=1-T_{1d},$$

where $T_{id}$ is the activity between 0 and 1 of TPV, the desired joint angle ($\theta_{d}$) is in the range ∈ [−π/2, π/2] and is based on the possible lengths developed by the artificial muscle. Indices $i=\left\{1,\;2\right\}$ indicate the agonist and antagonist terms, respectively. The biomimetic muscle in the experiments starts from the zero position (vertical position) where $T_{id}=0.5$ rad (see Figure 4).

The DV represents the activity of cells in area 5, and it is formulated as follows:

(A3) $$r_{i}=[T_{id}-x_{i}],$$

where $x_{i}$ is the PPV and represents the activity of cells in anterior area 5, and $r_{i}$ represents the activity of a DV cell.

The input from a decision center in the brain is the sigmoidal GO signal and is defined as follows:

(A4) $$\frac{d}{dt}g^{\left(1\right)}=\varepsilon \left[-g^{\left(1\right)}+\left(Cs-g^{\left(1\right)}\right)g^{\left(0\right)}\right],$$

(A5) $$\frac{d}{dt}g^{\left(2\right)}=\varepsilon \left[-g^{\left(2\right)}+\left(Cs-g^{\left(2\right)}\right)g^{\left(1\right)}\right],$$

(A6) $$g=g^{\left(0\right)}\frac{g^{\left(2\right)}}{Cs},$$

where g is the GO signal that multiplies the DV, ε is a slow integration rate, $g^{\left(0\right)}$ is the step input from a forebrain decision center, and Cs is the value at which the GO cells saturate.

The DVV describes the area 4 phasic movement time (MT) activity and is described by the following equation:

(A7) $$u_{i}={\eta gr}_{i},$$

where $u_{i}$ is the DVV, and η is the gain from the PPV to the OPV.

El OPV, or the average firing rate of a population of tonically active cells in area 4, is expressed as follows:

(A8) $$\frac{d}{dt}y_{i}=\mu \left[u_{i}+\left(x_{i}-y_{i}\right)\right],$$

where $y_{i}$ is the average firing rate of a population of area 4 tonic cells called OPV, and μ is a scaling factor for the integration rate.

The measured angular position of the artificial muscle is transformed and normalized ($L_{n}$) in the same way as Equations (A1) and (A2):

(A9) $$L_{1n}=\frac{\theta +\pi /2}{\pi },$$

(A10) $$L_{2n}=1-L_{1n},$$

where $L_{in}$ represents the current normalized length of muscle (*i*) within a range of 0 to 1 (e.g., $L_{in}=0.5$ when θ=0 rad).

The activity of muscle spindle primary afferent fibers (Ia) records changes in muscle elongation velocity and is modeled as:

(A11) $$s_{i}^{\left(1\right)}=\left[u_{i}-\frac{d}{dt}L_{in}\right].$$

Changes in muscle length *i* are recorded by secondary spindle afferents (II) and are given by:

(A12) $$s_{i}^{\left(2\right)}=\left[y_{i}-L_{in}\right].$$

Within the system, velocity errors from the primary spindle feedback are captured by an IFV, identified by phasic cell activity in reaction time (RT) area 4 [57], as described below:

(A13) $$q_{i}=\lambda s_{i}^{\left(1\right)},$$

where λ is the feedback gain.

The SFV plays an important role in static force compensation within the neural control system. This is achieved by integrating position errors detected by secondary spindle feedback and incorporating this information into the command of the alpha motoneuron. This process is described as follows:

(A14) $$\frac{d}{dt}f_{i}=hs_{i}^{\left(2\right)},$$

where h is a gain that controls the speed and strength of load compensation.

The PPV calculates the activity of tonic cells in anterior area 5, which receive position error feedback from muscle spindles and efferent copy input from area 4. This activity can be expressed as follows:

(A15) $$x_{i}=y_{i}-s_{i}^{\left(1\right)}-s_{i}^{\left(2\right)}.$$

The OFPV refers to the activity of phasic-tonic cells and expressed by the formula:

(A16) $$a_{i}=y_{i}+q_{i}+f_{i}.$$

The alpha motor neurons (α-MN) are presented as:

(A17) $$\alpha_{i}=a_{i}+\delta s_{i}^{\left(1\right)},$$

where δ is the gain of the stretch reflex.

The proposed cortical neural circuit generates its control actions through a dynamic muscle force model that emulates an elastic tissue and a neurally adjustable contractile element, giving it a neuronally adjustable threshold length for contractile force development. These muscle forces are transmitted to the triple-layered biomimetic muscle as agonist and antagonist control signals to regulate voluntary movement, similar to how living organisms would.

For simplicity, it can be posited that muscle force generation ($F_{i}$) is a linear threshold function of its length ($L_{in}$), depending on its stiffness (K), constant resting length ($\Gamma_{i}$), and contractile state, which can be subject to a neurally modifiable contractile state ($c_{i}$). To achieve a more accurate representation of the actual muscle, whose stiffness also changes depending on its contractile state, a quadratic force–length relationship is employed, including viscosity and non-linear stiffness. The dynamics of muscular force are depicted as follows:

(A18) $$F_{i}=K{\left({\left[L_{in}-\left(\Gamma_{i}-c_{i}\right)\right]}^{+}\right)}^{2}+F_{i0},$$

where $F_{i0}$ is a positive force and represents the initial prestress which has no effect on joint torque, and the notation ${\left[w_{i}\right]}^{+}$ is defined as $max(w_{i},\;0)$ so that if $w_{i}=L_{in}-\left(\Gamma_{i}-c_{i}\right)>0$, then $F_{i}=Kw_{i}$, whereas if $w_{i}\leq 0$, then $F_{i}=Kw_{i}=0$. Equation (A14) demonstrates that a muscle behaves similar to a spring, generating force only when it is stretched ($L_{in}$) beyond its effective threshold length $\left(\Gamma_{i}-c_{i}\right)$. Additionally, it also reveals that the muscle is more adaptable than a typical spring, as this threshold can be adjusted neurally by varying the muscle’s contraction state, $c_{i}$. The dynamics of the contractile state is expressed as:

(A19) $$\frac{d}{dt}c_{i}=\upsilon \left(c_{i}+\alpha_{i}\right)-{\left[F_{i}-\Gamma_{F_{i}}\right]}^{+},$$

where υ is contractile rate, $\Gamma_{F_{i}}$ is the force threshold in muscle control channel i*,* and $\alpha_{i}$ is the activity of the alpha motor neuron pool in muscle control channel i. In the case of voluntary motions of the joints, the system works through the involvement of area 4.

Agonist–antagonist forces ($F_{i}$) are control signals that are transformed into anodic and cathodic electrical currents ($T_{EC}$) that are sent to the corresponding layers of the three-layer artificial muscle. This transformation is obtained as follows:

(A20) $$F_{a1}=\left\{\begin{array}{cc} F_{1}\cdot sgn\left(\theta_{d}\right) & if\;\left|e\right|\geq 0.005\;\\ 0 & if\;\left|e\right|<0.005 \end{array}\right.$$

(A21) $$F_{a2}=\left\{\begin{array}{cc} F_{2}\cdot -sgn\left(\theta_{d}\right) & if\;\left|e\right|\geq 0.005\\ 0 & if\;\left|e\right|<0.005 \end{array}\right.$$

(A22) $$I_{i}=F_{ai}\cdot A_{ec}/1.125$$

where *e* is the error signal ($\theta_{d}-\theta$), $F_{ai}$ are rectified agonist–antagonist forces, $A_{ec}$ is the electrical current limit in mA, and $I_{i}$ are anodic and cathodic currents corresponding to each biomimetic muscle layer. Parametric values used in the cortical neural network and initial conditions in the experiments were Cs=25, ε=1, η=1, δ=0.1, h=0.025, λ=8.5, $g^{\left(0\right)}=2.0$, $g^{\left(1\right)}=0$, $g^{\left(2\right)}=0$, $y_{i}(0)=0.5$, $x_{i}(0)=0.5$, $a_{i}(0)=0.5$, μ=1, K=1.8, $\Gamma_{i}=0.95$, $F_{i0}=2.5\;N$, $\Gamma_{F_{i}}=10$, υ=0.25, and $A_{ec}=[1,\;12]$ mA.
